# Development of a data science CURE in microbiology using publicly available microbiome datasets

**DOI:** 10.3389/fmicb.2022.1018237

**Published:** 2022-10-12

**Authors:** Evelyn Sun, Stephan G. König, Mihai Cirstea, Steven J. Hallam, Marcia L. Graves, David C. Oliver

**Affiliations:** ^1^Department of Microbiology and Immunology, University of British Columbia, Vancouver, BC, Canada; ^2^Michael Smith Laboratories, University of British Columbia, Vancouver, BC, Canada; ^3^Graduate Program in Bioinformatics, University of British Columbia, Vancouver, BC, Canada; ^4^Genome Science and Technology Program, University of British Columbia, Vancouver, BC, Canada; ^5^Life Sciences Institute, University of British Columbia, Vancouver, BC, Canada; ^6^ECOSCOPE Training Program, University of British Columbia, Vancouver, BC, Canada

**Keywords:** data science, microbiome, amplicon sequencing, undergraduate education, course-based undergraduate experience

## Abstract

Scientific and technological advances within the life sciences have enabled the generation of very large datasets that must be processed, stored, and managed computationally. Researchers increasingly require data science skills to work with these datasets at scale in order to convert information into actionable insights, and undergraduate educators have started to adapt pedagogies to fulfill this need. Course-based undergraduate research experiences (CUREs) have emerged as a leading model for providing large numbers of students with authentic research experiences including data science. Originally designed around wet-lab research experiences, CURE models have proliferated and diversified globally to accommodate a broad range of academic disciplines. Within microbiology, diversity metrics derived from microbiome sequence information have become standard data products in research. In some cases, researchers have deposited data in publicly accessible repositories, providing opportunities for reproducibility and comparative analysis. In 2020, with the onset of the COVID-19 pandemic and concomitant shift to remote learning, the University of British Columbia set out to develop an online data science CURE in microbiology. A team of faculty with collective domain expertise in microbiome research and CUREs developed and implemented a data science CURE in which teams of students learn to work with large publicly available datasets, develop and execute a novel scientific research project, and disseminate their findings in the online Undergraduate Journal of Experimental Microbiology and Immunology. Analysis of the resulting student-authored research articles, including comments from peer reviews conducted by subject matter experts, demonstrate high levels of learning effectiveness. Here, we describe core insights from course development and implementation based on a reverse course design model. Our approach to course design may be applicable to the development of other data science CUREs.

## Introduction

Advances in sequencing throughput and mass spectrometry are rapidly converting biology into a data-driven science in which multi-dimensional datasets contribute to knowledge at the individual, population and community levels of biological organization (Higgs and Attwood, 2005; Hahn et al., 2016). While multi-dimensional data generation in life sciences research becomes normative, working with these complex datasets to answer scientific questions with meaning and insight remains challenging across training levels, and raises the question of how to prepare undergraduate students in particular for data-driven research based on scaffolding and development of core competencies (Attwood et al., 2019; Irizarry, 2020).

One way to approach this challenge is to leverage existing pedagogical frameworks that embed authentic research experience in undergraduate teaching and learning. Course-based undergraduate research experiences, known as CUREs, are scalable, broadly accessible, credit-based courses where students conduct authentic research projects often in team-based settings. Auchincloss et al. (2014) have proposed that CUREs encompass core research competencies, including scientific practices, collaboration, iteration (as experiments, ideas and hypotheses are refined), discovery, and relevance as the research topics are novel and have meaning beyond the walls of the classroom. As such, several curricular innovations have emerged over the last decade that explore data science through CUREs (Wang, 2017). Furthermore, remote learning due to the global COVID-19 pandemic prompted a recent surge in undergraduate lab curricula pivoting from a “bench-based” or “wet lab” research perspective to a computational (dry-lab) one. Supplementary Table 1 captures some of these educational innovations spanning the central dogma of biology from DNA > RNA > proteins > metabolites. Just as life science has become a multi-omics experience expanding its focus from DNA sequencing (genomics) to other forms of biological information (e.g., transcriptomics, proteomics, metabolomics), so have many new CUREs. However, the emerging data science CUREs in 2008–2009 emphasized more conventional software tools such as implementing the Basic Local Alignment Search Tool (BLAST; Furge et al., 2009; Lau and Robinson, 2009) for database searches or ClustalW or ClustalX for multiple sequence alignment (Campo and Garcia-Vazquez, 2008; Furge et al., 2009). In contrast, recent CUREs implement more programmatic approaches to using software tools that involve data wrangling and statistical inference including correlation networks (Brown, 2016), gene expression (Makarevitch et al., 2015), and microbial community profiling (Sewall et al., 2020; Zelaya et al., 2020; Baker et al., 2021).

Including a wet-lab component in a data science CURE in which students first generate *de novo* datasets provides an exceptional learning context for authentic research. However, this model can pose logistic, temporal and financial barriers that can limit efficacy and sustainable adoption. First, datasets will likely be constrained due to limited time allotted for experimentation as well as access to essential infrastructure and sequencing resources. This puts added pressure on students to generate useable data while their experimental skills are still under development. The resulting datasets will also be limited in scope thus constraining the types of analysis that can be performed and the biological questions that can be answered. Finally, *de novo* data generation limits the time available for developing data science skills needed to perform analyses. Based on these constraints, a data science CURE that leverages public datasets as teaching and learning resources could provide a more tenable model. Here we describe such a course combining the structure of a previously established wet-lab CURE (Sun et al., 2020a) and modular data science curriculum developed in the context of the Experiential Data Science for Undergraduate Cross-disciplinary Education (EDUCE) initiative (Dill-McFarland et al., 2021). We describe core insights from course development and implementation based on a reverse course design model using small subunit ribosomal RNA (SSU or 16S rRNA) gene sequences sourced from public datasets with emphasis on extensibility and adoption within the broader CUREs teaching and learning community.

## Course design

Since 2001, the Department of Microbiology and Immunology at the University of British Columbia in Vancouver, BC, Canada, has been implementing a wet-lab CURE model centered around student publications in an undergraduate research journal called the Undergraduate Journal of Experimental Microbiology and Immunology (UJEMI; Sun et al., 2020a,b). In brief, student teams design their research projects inspired by the research published by their peers in UJEMI. The skills and domain knowledge required to generate an original UJEMI manuscript define the learning outcomes for this CURE model as summarized in Table 1.

**Table 1:** General and technical course learning objectives aligned to the domains of a CURE as defined by Auchincloss et al. (2014).

Learning objectives	Domain of a CURE if relevant
By the end of this course, students will be able to: Overarching objective: Apply science process skills to address a research question in a course-based or independent research experience.	All domains
**General scientific development (adapted from** Clemmons et al., 2020**):**	
1. Explain how science generates knowledge of the natural world.	Scientific practice
2. Locate, interpret, and evaluate scientific information.	Scientific practice, broader meaning
3. Pose testable questions and hypotheses to address gaps in knowledge.	Scientific practice, iteration, discovery, broader meaning
4. Plan, evaluate, and implement scientific investigations.	Scientific practice, iteration
5. Interpret, evaluate, and draw conclusions from data in order to make evidence-based arguments about the natural world.	Scientific practice, iteration
6. Work productively in teams with people who have diverse backgrounds, skill sets, and perspectives.	Collaboration
**Technical development:**	
7. Connect to and work in a server environment using command line.	
8. Maintain an annotated record of programming scripts.	Scientific practice: documentation
9. Describe the different steps of the QIIME2 pipeline.	
10. Adapt the QIIME2 pipeline to different datasets.	
11. Interpret and analyze microbiome data.	
12. Perform microbiome analyses using R and RStudio.	
13. Generate and interpret alpha and beta diversity outputs.	

General scientific development learning objectives were adapted from Clemmons et al. (2020).

In 2020, with the onset of the COVID-19 pandemic and the shift to online teaching, we set out to build an alternative data science CURE model in which students plan a research project using public data, conduct data processing and analysis steps, and disseminate their findings (Sun et al., 2020a). Design of this new course involved (1) vetting the scope and breadth of research projects, (2) leveraging the existing CURE model to build pedagogical scaffolding to provide students with the skills required to carry out their projects, and (3) assembling resources such as domain expert teaching assistants. As a first step, research faculty and educators with expertise in data science joined the core CURE design team to assemble the necessary domain knowledge to form a course development team.

### Vetting the scope and breadth of research projects

#### Data type selection

**Figure 1:**
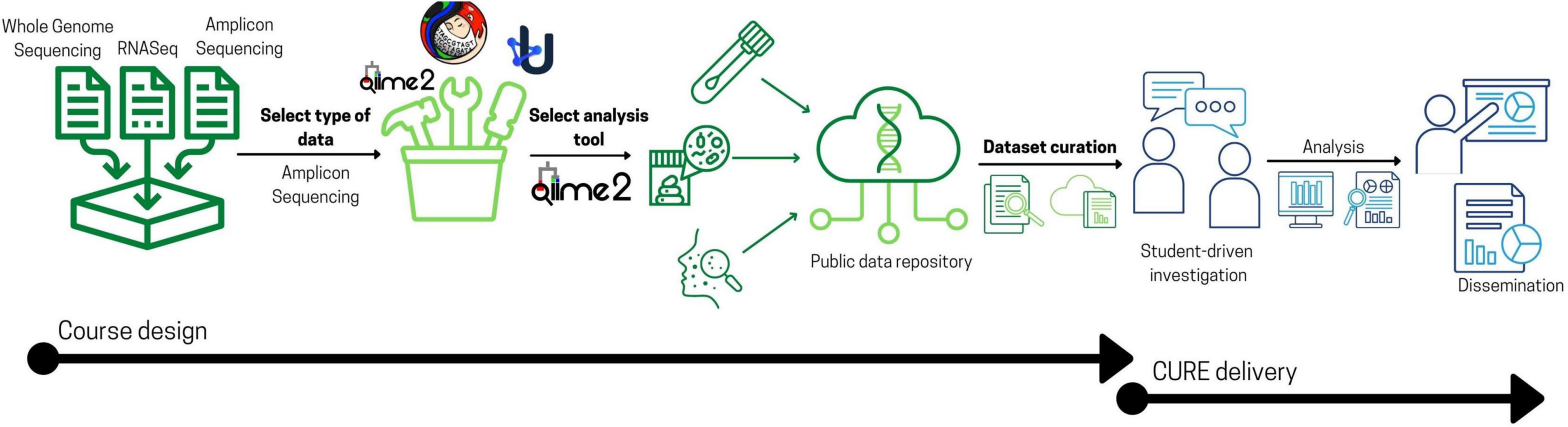
Core decision points of course design involved in vetting the scope and breadth of research projects. Designing a data science CURE from our perspective started with narrowing the scope of the projects by: (1) deciding on the type of data to focus on where in our case, we selected amplicon sequencing data; (2) deciding on a particular tool to teach, we selected QIIME 2, and (3) ultimately curating datasets from public repositories where research groups around the world contribute to. The implementation phase (detailed in Figure 2) involves student-driven investigations and analyses that are disseminated into an undergraduate research journal, UJEMI.

The course was developed iteratively through a series of discussions focused on the types of research projects to be supported including data sources and types, software applications, and analysis methods. Initially we considered allowing students to work with any type of biological data spanning the central dogma. However, the large variety of analyses in this model would have fragmented the instructional effort to a degree deemed unfeasible in a relatively high enrollment CURE (e.g., greater than 50 students). Furthermore, students in our program enter the course with limited prior experience related to data-driven analysis. For these reasons, the team decided to constrain the course to (1) a single type of data and (2) an integrated software framework for data processing and analysis (Figure 1). The team considered several types of biological data, including amplicon sequencing, genome assembly, and RNA-seq based on the potential for student development, alignment with our undergraduate curriculum, required scaffolding, and practical relevance. Ultimately, we decided to focus course projects on using 16S rRNA gene amplicon sequences as a robust introduction to data science in microbiology.

Amplicon gene sequencing is a DNA-based method that involves PCR amplification and sequencing of a specific genomic interval. In the context of microbiology, this interval is most commonly one or more variable regions in the 16S rRNA gene. It is used to describe microbial communities within a sample (e.g., feces, soil, skin; Cullen et al., 2020). It essentially resolves taxa present in the community and allows for both compositional and ecological diversity analyses. Current sequencing platforms can generate thousands of amplicon sequences per sample enabling more quantitative insights into microbial community structure. Each sequence variant within a sample essentially represents a bacterial taxon, resolvable across ranks from phylum to species using conventional hierarchies. The concept of 16S rRNA gene amplicon sequencing is simple enough for a course introducing students to data science, but also allows relatively complex projects on topics of broader interest.

Additional criteria supporting our decision included: (1) the underlying concepts of 16S rRNA gene sequence analysis are well established in our undergraduate curriculum, e.g., consideration of its ancestral role in information processing, relevance to phylogenetic inference including the discovery of the 3rd domain of life, as well as concepts related to microbial diversity (Woese and Fox, 1977), (2) amplicon sequencing technology is widely employed to study microbial community structure, e.g., microbiome composition in natural and engineered environments including our own bodies, and (3) extensive research activity in this area over recent years has generated many large datasets that have been made publicly available with metadata that have not been fully investigated.

#### Integrated software framework

Having decided to use amplicon sequencing data, we set out to identify an integrated software framework to support student training and ongoing project development (Figure 1). Among established software used for this application we settled on QIIME 2 (Bolyen et al., 2019) due to the availability of extensive tutorials, online community support, and widespread adoption by industry and academic research labs. A key pedagogical reason for selecting QIIME 2 is that the analysis begins with simple universal steps and increases in complexity. The first step is simply a copy-paste command and only requires that the students can navigate a server. Students then view and interpret the output to adjust a single analysis parameter for their second step. Later, more complex decisions are necessary to choose among different diversity metrics whose results entail more sophisticated interpretation while still using the standard QIIME 2 interface. Finally, students move into R, a language and software environment for statistical computing and data visualization, for more creative and refined analyses that require more complex interpretations. The progression from the QIIME 2 web interface to command line into R involves a progressive scaffolding process that builds core competency in data science through the lens of 16S rRNA amplicon sequences, and requires students to apply more compounded levels of thinking as the course progresses.

#### Dataset acquisition and curation

The next task was to acquire datasets suited for novel analysis by novice users using QIIME 2 and R. Initially, we searched locally and solicited UBC researchers for data, but this turned out to be more difficult than expected, and we were unable to source more than one useable dataset. To broaden the search for appropriate data sources we hired a domain expert teaching assistant to curate datasets from published papers. The datasets were scrutinized and ranked as suitable for student projects based on the following criteria: (1) availability of “unmined” metadata (independent variables that were not fully explored in previous publications involving the dataset), (2) data is complete (all samples and categories are available as mentioned in the original publication), (3) sufficient sequence quality (data yields reliable results). The term metadata describes independent (i.e., controlled) variables recorded by the primary researcher describing each sample, for example, host age, sex, geographical location of sample collection site, or diet. Metadata might be immediately relevant for the initial study’s design or considered for future studies. An investigator might only strictly explore a microbiome dataset and the associated metadata to answer previously defined research questions leaving other metadata unexplored. In some cases, datasets are incompletely analyzed and are made available to other researchers following deposition in publicly accessible online repositories. The number of variables in the metadata were expected to define the longevity of the dataset in the course, where more variables support more diverse research questions over time.

Using personal computers, students remotely accessed a server environment provisioned with sufficient memory and computing power. The server acted as a virtual lab to process, manage and analyze data. Due to the size and the amount of computational power or time necessary to process the datasets, students were provided with computational resources (i.e., access to the remote server) ensuring equitable working conditions. A departmental IT expert was essential to set-up and maintain server resources throughout the course.

### Adapting the CURE model

Our wet-lab CURE follows a 16-week research cycle divided into 3 phases: planning, experimentation, and dissemination (Sun et al., 2020a). As a capstone course, students enter the CURE with an established foundation of microbiology skills and concepts and design their research questions based on a body of published student work in our in-house undergraduate journal, UJEMI. The journal thereby acts as a repository of student-authored data that drives the investigative direction of incoming students.

**Figure 2:**
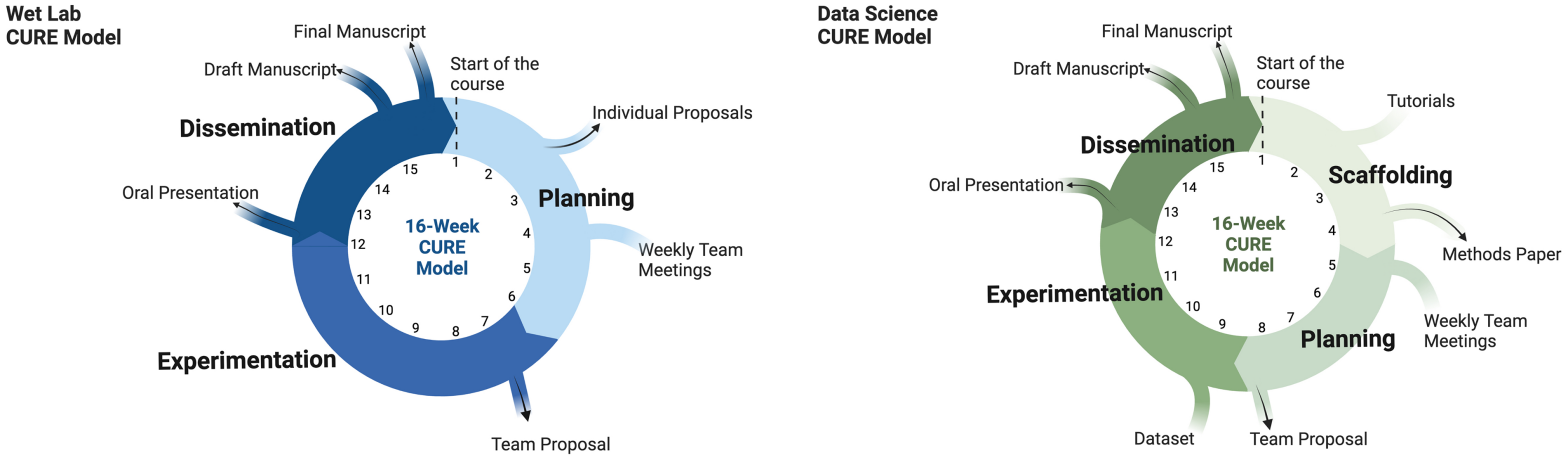
3-phase wet-lab CURE model compared to 4-phases of the data science CURE. A scaffolding phase was added to the data science CURE. During the scaffolding phase, students use a combination of tutorials and lectures to learn about amplicon sequence analysis through the use of QIIME 2. Subsequent phases are similar in nature between the wet lab and data science CURE. The planning phase involves teams of 3 to 4 students developing a novel research question(s) and developing a proposal on how to answer said question(s). Proposed analyses are conducted during the experimentation phase which is done on a remote server environment (i.e., a remote computer that can be accessed on a personal computer) or in the lab. Students present and write up their findings during the dissemination phase. All student projects are published in the Undergraduate Journal of Experimental Microbiology and Immunology (UJEMI).

We recognized that students entering the data science CURE would have minimal to no data science experience making additional scaffolding necessary. In our wet-lab CURE, students enter the course having completed a set of prerequisite wet lab courses. In contrast, preliminary student survey data in the data science CURE (Supplementary Figure 1) indicated that only about half of the respondents had some previous data science exposure, usually from other undergraduate courses. Our philosophy was that students should have a concrete understanding of how biological samples are processed to collect genetic information as digital data and then used to produce statistical results and visualizations. Based on these skills and the domain knowledge required to generate an original research article suitable for publication in UJEMI, we defined learning outcomes (Table 1) and used reverse course design to develop classroom activities and assignments to scaffold student learning. The resulting data science CURE was divided into two phases over 16 weeks: the scaffolding phase and research investigation phase, the latter followed by the same three stages as our original wet-lab CURE (Figure 2). We found this to be a significant difference from our wet-lab model, where students start planning their investigations from the outset of the term. This change could be accommodated because the data science projects were feasible within a relatively short time frame compared to some of the wet-lab projects (see section Course implementation for an outline of our model).

### Assembling the teaching team

Once the course structure was defined our attention shifted to implementation with particular emphasis on teaching team composition. Experienced data scientists and CURE instructors co-taught the course, a teaching model well established in the literature as an effective means of promoting learning (Gillespie and Israetel, 2008; Chanmugam and Gerlach, 2013). In collaboration with the CURE instructors, experts in data science developed content fitting the CURE model, such as data wrangling and analysis, workflows, experimental logic and specific aspects of project design. The CURE instructors’ limited experience in data science was beneficial as their beginner’s mindset facilitated content design at a level appropriate for new learners ensuring students understood not only what they were doing but why.

We recruited domain expert graduate student teaching assistants (TAs) to help support the development and implementation of the course. TAs were selected from within and outside of our home department as graduate students who work extensively with amplicon sequencing data as part of their thesis projects. TAs often had prior experience as teaching assistants but no formal pedagogical training. TAs were extensively involved in the curriculum development process that occurred before implementation of this course including creating content to scaffold student learning and developing tutorials to manage tools and datasets.

Weekly student team meetings with TAs and instructors were integral to successful implementation of this CURE. These meetings were used to discuss project development, analyze data and sort out team dynamics. TAs contributed both mentorship and expertise. TAs with more domain expertise also provided guidance and training when student projects evolved beyond the core analyses introduced in lectures to pursue more refined analyses, ones that we termed “boutique analyses.” Outside of the core course curriculum, students pursued boutique analyses to address specific aspects of their research questions. On average, each TA was responsible for mentoring 4 to 5 teams per term which equated to approximately 3 to 4 h of student meetings per week. During the first iteration of the course with 60 students, 2 instructors were supported by 4 TAs.

## Course implementation

Since developing this course in 2020, we have been offering it in the Fall (September–December) and Winter (January–April) terms. The CURE serves approximately 40–60 students per term. This section provides an overview of how the course operates in each of the four phases of our data science CURE (Figure 2).

### Scaffolding phase (week 1–4)

The course begins with the scaffolding phase, where students learn the core concepts and basic coding skills underpinning amplicon sequence analysis in lectures essential to their project. In this phase all assessments are assigned to each individual student. The last three phases of the CURE are completed as a team, and students are assessed as a team. We identified three critical areas for learning, including (1) understanding the biochemistry and molecular biology involved in converting a sample containing microbes to digital sequence information, (2) understanding basic concepts required to interpret ecological diversity metrics and (3) the skills required to work with the selected software framework. We make use of existing tutorials published on the QIIME 2 website (Bolyen et al., 2019) to reinforce core concepts covered in lectures. We conclude this phase by implementing individual student assessments which include a quiz and short assignment where students write a technical paper on the QIIME 2 pipeline which addresses Learning Objectives (LOs) 9–11 (Table 1).

### Planning phase (week 5–8)

Publicly available datasets are introduced into the course as the starting point for student investigations during the planning phase (Figures 1, 2). Students form project teams of three to four participants. Student teams discuss their projects in weekly meetings with teaching team members. All meetings are conducted synchronously. Similar to our wet-lab CURE, students analyze the literature and the metadata associated with their selected dataset and pose novel research questions not addressed in the original published study. The planning phase culminates with submission of a team-based proposal describing the research project background, research objectives, hypothesis, workflow, and possible modes of analysis. The teaching team reviews the proposal and provides extensive feedback in both written and verbal forms to each team (see rubric in Supplementary material).

### Experimentation phase (week 9–12)

During the experimentation phase, students are responsible for independently scheduling the time spent on their project outside the regular course activities and distributing tasks among team members. Data processing is executed in a team-shared server environment, which plays a role similar to an open lab in the original wet-lab CURE model. Teams document their progress in shared lab notebooks in a format of their choice (often a shared drive file). In an informal in-class survey, most students reported spending on average 5 to 6 hour per week working on their projects (most likely fewer hours in the early stages of their project and more hours during late stages) in addition to the scheduled course activities which make up about 2.5 to 4 hour per week. Student workload (i.e., time commitment) in our data science CURE is approximately equivalent to our wet lab CURE.

### Dissemination phase (week 13–16)

In the final phase of the course, students disseminate their project findings first as an oral presentation to their peers and then as a full written manuscript. Teams first submit a draft manuscript and, after review by the instructor and teaching assistant (see rubric in Supplementary material), implement any feedback into their final manuscript. Final manuscripts are intended to be as publication-ready as possible and ultimately published in the undergraduate research journal, UJEMI. Students receive instructions on submitting their manuscript to the UJEMI editorial team for publication after course completion as either a non-referred or peer-reviewed article (Sun et al., 2020b). Of the 22 teams that participated in this CURE in September to December 2020 and January to April 2021, 8 teams decided to submit their manuscripts for formal review and publication in the peer-reviewed issue of UJEMI, UJEMI+. Manuscripts from the first two iterations of the course of non-referred^1^ and peer-reviewed articles^2^ can be found online.

## Outcomes

We collected data from the first 2 iterations of the course in September to December of 2020 (Term 1) with 60 students and January to April 2021 (Term 2) with 18 students. The collection of student data in this study was approved by the University of British Columbia’s Behavioral Research Ethics Board (Project ID: H19-02879). Students were divided into teams of 3 to 4 for a total of 16 teams and offered the choice of among 5 available datasets in term 1. In term 2, students were divided into 6 teams of 3, each assigned to a different dataset. The two iterations of the course were taught by two different instructors. We collected the following data to validate the model:

- Analysis of student manuscripts
- Peer reviews of manuscripts submitted to UJEMI
- Student perspective data

### Analysis of manuscripts

We assessed the scientific practice of students by analyzing the written course outputs from the first iteration. Fundamentals of this practice have been defined as collecting and analyzing data, disseminating scientific findings, contextualizing findings to the broader literature, collaborating with other researchers, and designing a research investigation (Lopatto, 2003; Buck et al., 2008; Weaver et al., 2008; Auchincloss et al., 2014) which align to our course learning objectives (Table 1 Learning Objectives (LOs) #2–6) and how student manuscripts were evaluated (see manuscript rubric in Supplementary material). We evaluated written proposals as evidence for designing an investigation (LOs #3,4) and final manuscripts as evidence for disseminating research findings (LO #5; Table 2). On average, teams cited 17 references in their proposal and 37 in their final manuscript showing relevance of their research topic within the broader literature. Each manuscript had, on average, 5 data-driven figures. Students referred to the broader literature (i.e., peer reviewed papers outside of the course) in the discussion section of their manuscripts to contextualize their findings. Citing an average of 6 papers, students reported corroboration, or in some cases contradiction, with their own data demonstrating balanced and rigorous scientific interpretation of their results.

**Table 2:** Summary of literature cited, data figures/tables and literature used to contextualize their own findings based on course outputs.

		Literature cited			
Project #	Dataset	Proposal	Final manuscript	# Data figures/tables	# Papers that students contextualized to their own findings
1	Organic matter removal treatment of soil (Wilhelm et al., 2017)	9	23	5/1	5
2		10	52	5	10
3		16	25	5	6
4		34	50	3	2
5	Infant feeding study (Dawson-Hahn and Rhee, 2019)	12	19	7	0
6		17	43	3	4
7		35	43	8	7
8		7	33	5	11
9	Human Parkinson’s study (Cirstea et al., 2020)	38	37	4/1	10
10		15	56	5	6
11		19	42	5	7
12		10	37	5	9
13	Hunter-gatherer lifestyle of the Hadza people of Tanzania (Smits et al., 2017)	16	32	4/2	4
14		17	34	7	7
15		10	56	5	6
16		6	23	5	2
17	Dog IBS study (Vázquez-Baeza et al., 2016)	7	22	5	7
18		13	47	4/2	6
19	Effects of animal captivity (McKenzie et al., 2017)	18	36	5	5
20		22	32	3	7
21		17	35	6	8
22	HI-SEAS space isolation study (Mahnert et al., 2021)	16	42	4	2

Literature cited was taken from the reference list at the end of each project proposal and final manuscript. The number of data figures/tables and the number of papers that students referenced in the discussion section that either corroborated or contradicted their findings were taken from their final manuscript. Data in this table was collected from 22 student projects spanning two iterations of the course offered in September–December 2020 (60 students, 16 teams of 3–4) and January–April 2021 (18 students, 6 teams of 3) where 7 different datasets were available to choose from.

Students selected different analyses (Table 3) indicating that a range of analyses were supported by the material used to scaffold the CURE. Most, if not all, teams analyzed core metrics taught in the lecture component. This included alpha- and beta-diversity, taxonomic assignment, and differential abundance, where the ability to generate diversity metrics was defined as a final course learning objective (LO #13). This final learning objective was supported by the technical learning objectives. Table 3 shows that student teams performed this analysis and generated the output showing that they had achieved the technical learning objectives of the course. Beyond this expectation, teams also conducted boutique analyses, including statistical tests specific to certain metadata types, as well as trait-based mapping (the others listed in Table 3) driven by their own initiative with support from instructors and TAs.

**Table 3:** Summary of analyses conducted per project and dataset.

		Analyses conducted								
Project #	Dataset	Alpha and beta diversity	Taxonomic analysis	Differential abundance	Correlation analysis	Linear regression	Logistic regression	Longitudinal analysis	Functional microbiota profiling	Total analyses conducted
1	Organic matter removal treatment of soil (Wilhelm et al., 2017)									3
2										2
3										3
4										4
5	Infant feeding study (Dawson-Hahn and Rhee, 2019)									3
6										3
7										3
8										2
9	Human Parkinson’s study (Cirstea et al., 2020)									3
10										3
11										3
12										3
13	Hunter-gatherer lifestyle of the Hadza people of Tanzania (Smits et al., 2017)									2
14										2
15										3
16										3
17	Dog IBS study (Vázquez-Baeza et al., 2016)									3
18										3
19	Effects of animal captivity (McKenzie et al., 2017)									2
20										3
21										3
22	HI-SEAS space isolation study (Mahnert et al., 2021)									2

Summary of analyses conducted per student investigation was derived from the methods section of the students’ final manuscripts. Students were taught alpha and beta diversity, taxonomic analysis, and differential abundance analysis (green) in class but only required to conduct an alpha and beta diversity analysis for their final project (dark green). All additional analysis (yellow) were “boutique analyses” that students pursued to expand the breath of their study including seeking out additional training and support for.

### Peer review

Eight teams out of the 22 from the first two iterations of the course (September 2020 and January 2021) chose to have their manuscripts published as peer-reviewed articles in UJEMI+ (Sun et al., 2020b). The feedback provided by domain experts who reviewed these student papers contributed interesting insight into the quality of research conducted by our students in comparison to real-world practices. Reviewers provided feedback on all aspects of the manuscripts. Comments often focused on missing details and rationale in Methods sections, and suggested authors provide more concise and careful interpretations in the discussions. Other suggestions emphasized the need for clarity in the presentation of figures and figure legends.. Most reviews indicated that the quality of research conducted by the students was considered to comply with “industry-standards.” Many of the more critical comments focused on the structure or writing of the manuscript rather than the depth or breadth of analysis reinforcing the effectiveness of the CURE model to support effective knowledge transfer and practice through course outputs. This validated that the expectations and standard of quality (see rubric for the manuscript in Supplementary material) we set for the final manuscripts reflect industry-standard practices.

### Student perspective data

In addition to the analysis of course outputs, we also implemented an end of course survey to gather insight into student perceptions of learning. This survey consisted of three parts: (1) a section on previous experience in research and data science (Supplementary Data), (2) the laboratory course assessment survey (LCAS; Corwin et al., 2015), (3) questions about internal and external collaborations. The survey was implemented in the first 2 iterations of the course (September to December 2020, January to April 2021) with a response rate of 45% (n = 35).

Results from the first section of the survey which asked students about their prior experience in bioinformatics (Supplementary Figure 1) indicated that all respondents (*n* = 27) had previously participated in an undergraduate research experience (URE) at some point in their degree, most during the latter half. The experiences ranged from volunteer experiences to full-time paid internships, also called co-ops (summary in Supplementary Figure 1). Among the respondents, 52% indicated that they had some data science experience before the course. Most of them attributed this experience to previous courses in the program participating in the EDUCE initiative (Dill-McFarland et al., 2021), and a few to their previous UREs. In total, 65% of the students indicated that they were in a team with at least one student with prior data science experience and felt this was helpful in moving projects forward. Among students without any team members with previous experience, 60% considered it a disadvantage.

To further assess learning effectiveness, we administered the LCAS, a validated, well-established survey (Corwin et al., 2015). The LCAS measures student perceptions of participating in collaboration, broader discovery, and iteration in terms of frequency and challenge. We gathered 18 responses (30% response rate) in term 1 (September to December 2020) and 17 responses (94% response rate) in term 2 (January to April 2021). The two iterations of the course were taught by different instructors, but we did not observe significant differences in responses between the two terms suggesting no instructor bias. The data was combined for subsequent analysis.

**Figure 3:**
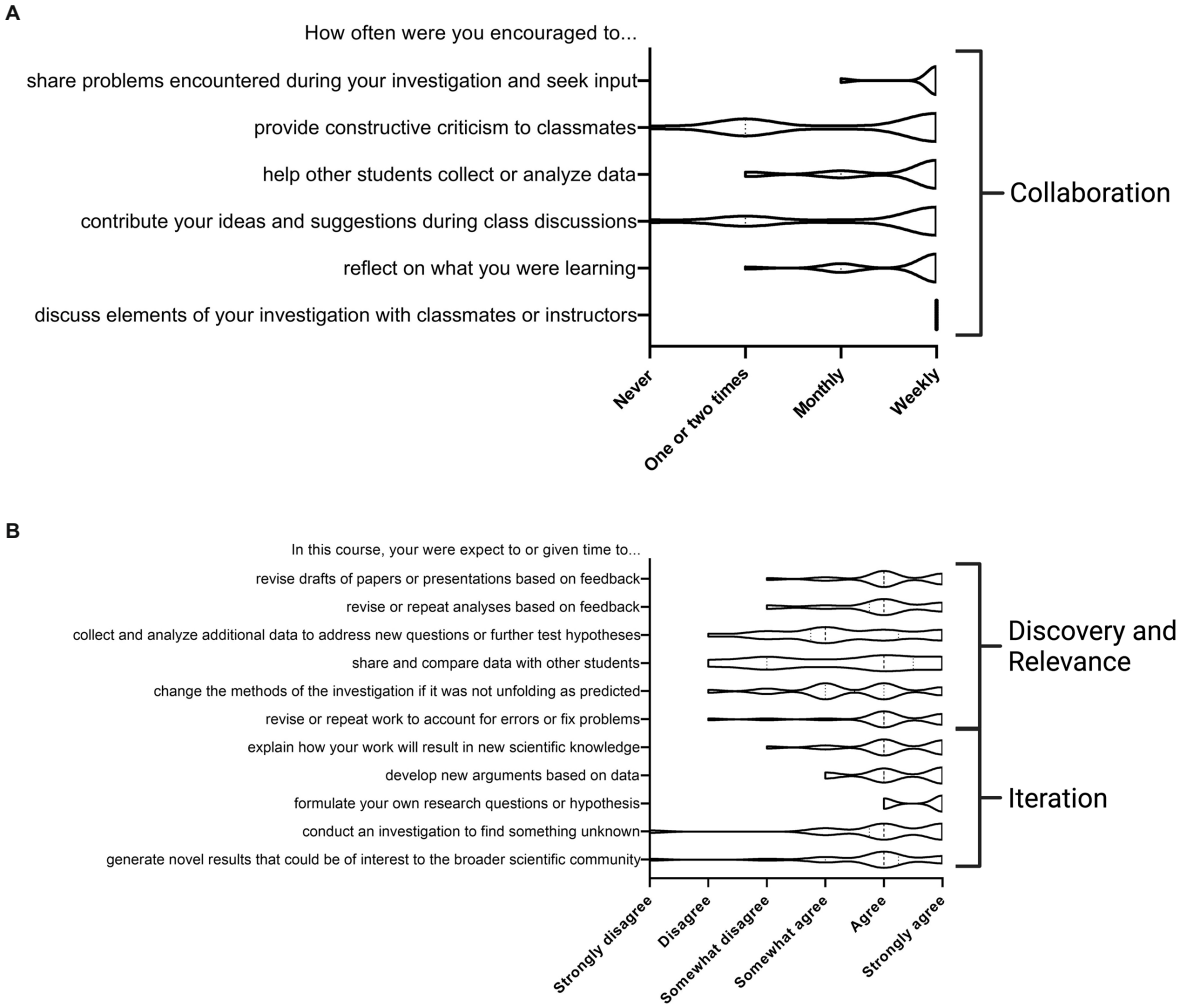
Laboratory course assessment survey (LCAS) results supported student perceptions that they participated in collaboration, iteration, broader meaning, and discovery. Students were asked about the frequency of activities **(A)** and course expectations **(B)** that align to 4 of the 5 domains of a CURE as indicated above with discovery and relevance grouped together. The above data represents 35 responses out of 78 students that took the course in September–December 2020 and January–April 2021. This survey was developed by Corwin et al. (2015).

Based on the LCAS data (Figure 3), most respondents agreed that they covered the content indicated in the course manual which aligns with the core learning objectives for the CURE. All the responding students reported frequently discussing their investigation with their peers, instructors and TAs. They did not think that they often participated in providing constructive feedback to their peers, which may be an area for further improvement. From the survey data we were able to identify at least 2 cases of inter-team collaboration and 1 of external collaboration that occurred during the two instances of this course. Teams collaborated to share analysis resources or information from external sources. One case of external collaboration happened when a team sought support from experts in the field. Promoting student collaboration is an area of focus for future iterations of the course.

Doing research tends to be time intensive and students in our wet-lab CURE report that they invest approximately 6 to 8 hours per week on the project which includes lab work, team meetings, and lectures. Students in our dry-lab CURE report a similar time investment; however, the computational nature of the work provides added flexibility as students can work remotely and outside of the hours that would be allocated for wet-lab experimentation (e.g., 8 a.m. to 5 p.m. weekdays). Data science workflows also lend themselves to more rapid processing and iteration (e.g., minutes to hours) compared to the wet-lab where repeating an experiment can take days to weeks. These attributes associated with a data science CURE (e.g., flexibility, remote work, rapid iteration) are well suited to students requiring approaches to education where personal constraints exist (e.g., commuting students, family obligations, work requirements, living in off campus rural locations).

## Discussion

Here we describe the development and implementation of a new data science CURE that leverages existing, published 16S rRNA gene amplicon sequencing datasets to study microbial community structure. Many new data science-driven CUREs have emerged in the last 2 years, especially in microbiome research (Jung et al., 2020; Sargent et al., 2020; Sewall et al., 2020; Zelaya et al., 2020; Baker et al., 2021). Emerging CURE models in this area have focused on student-generated datasets coupling a dry-lab experience with a wet-lab component. We decided to forgo a wet-lab experience and focus exclusively on data processing and analysis using public datasets. This approach allowed us to concentrate primarily on developing core competencies in data science while exposing the students to real-world data in the context of a CURE (16 weeks).

Based on our experience we explain (i) our rationale for using 16S rRNA gene amplicon sequencing analysis in our CURE, (ii) how our data science CURE aligns with the 5 proposed domains of a CURE (Auchincloss et al., 2014), and (iii) key considerations in the design of a data science CURE.

(i) Using 16S rRNA gene amplicon sequences provides a robust introduction to data science in microbiology for the following reasons:

**Rich data source for novel research:** Microbiome studies are of broad interest with exciting and dynamic research potential (Cullen et al., 2020) and readily available in large public dataset repositories. Datasets are often underexplored, allowing students to devise and pursue novel research questions within the constraints of the course timeline.

**Pedagogical advantages:** The workflow for 16S rRNA gene amplicon sequence analysis provides a framework for learning. Analysis starts with a reasonably simple processing step offering a more accessible point of entry for students with minimal to no data science experience and develops into more complex analyses and decision points. The uniform structure of these data enables a standard workflow and the sequence diversity requires critical thinking at each analysis step.

**Low cost:** The software used for 16S rRNA gene amplicon sequence analysis (QIIME 2 and R) in this CURE are free and well documented [https://docs.qiime2.org/2021.8/; (Bolyen et al., 2019)]. The size of these datasets is reasonably small (usually several megabytes per sample), reducing the demand on university servers and allowing for rapid command execution.

(ii) Our course model aligns with the five proposed CURE domains (Auchincloss et al., 2014) as follows:

**Scientific practices:** Each team develops a novel research question, designs and executes experimental workflows, and reports their research findings as an oral presentation and published manuscript.

**Discovery:** Students pursued novel research questions and generated data to analyzed and gather new insights.

**Collaboration:** Students work in teams of 3 to 4 and conduct research on datasets generated by research groups from around the world. In some instances, student teams collaborate within the classroom as well as with researchers outside of the classroom who had generate the primary data.

**Iteration:** Weekly team meetings offer students the opportunity to refine their research questions and troubleshoot methods. Student teams use feedback received on end-of-term oral presentation and draft manuscript to revise the final manuscript for publication.

**Broader meaning:** Student teams discuss their results in the context of other published studies. Comments from peer review have consistently indicated that the students’ research findings were of general interest to the broader scientific community.

(iii) Based on our experience, the following requirements were essential to the development and implementation of our data science CURE model, which may be useful to other educators developing similar courses:

- Assembling an effective team of both domain and educational experts.
- Constraining the type of data and software used by the students in their projects.
- Acquiring resources such as datasets from publicly available databases, a computational framework, and expert teaching assistants.
- Developing scaffolding teaching material around the type of data and tools used.

Our model for a data science CURE is both sustainable and scalable. Students publish their findings in our in-house journal, UJEMI, creating an archive of student-authored projects which minimizes project repetition and primes the direction of novel research projects. To sustain this model, we anticipate introducing new 16S rRNA gene amplicon sequence data sets into the course every 2 to 3 years to refresh and seed new course projects. For future iterations of the course, we will continue to work with published datasets and add additional ones from the microbiome research community. Establishing and fostering connections between our students and active research groups around the world is a program goal. At present, our data science CURE accommodates approximately 60 students per term; however, we can envision scaling up the course size given the necessary teaching resources. Unlike a wet lab CURE requiring lab space and equipment, our data science CURE uses personal computers and servers so “experiments” in the form of computational workflows can be done in regular classrooms or remotely. Enrolment in our data science CURE is primarily constrained by the availability of experienced graduate student teaching assistants. We anticipate that teaching assistant expertise will become more readily available as the field develops and more students receive data science education, in courses such as this one, as well as graduate school. Skills acquired through data science CUREs will serve students well as demand for scientists with domain knowledge (e.g., microbiology) combined with data science experience grows.

## Data availability statement

The original contributions presented in the study are included in the article/Supplementary material, further inquiries can be directed to the corresponding author.

## Ethics statement

The studies involving human participants were reviewed and approved by University of British Columbia’s Behavioral Research Ethics Board (Project ID: H19-02879). The participants provided their written informed consent to participate in this study.

## Author contributions

Conceptualization and acquisition of funding was performed by DO, MG, and SH. Data collection and analysis was conducted by ES. Original draft was prepared by ES, SK, and MC with refinement by SH. All authors contributed to the article and approved the submitted version.

## Funding

Funding for the work presented in this manuscript was provided by the University of British Columbia’s Department of Microbiology and Immunology and a grant awarded by UBC’s Program for Undergraduate Research Experience (https://research.ubc.ca/about-vpri/program-undergraduate-research-experience-call-proposals/pure-funding-recipients) to DO, MG, and SH.

## Conflict of interest

SH is a co-founder of Koonkie Inc., a bioinformatics consulting company that designs and provides scalable algorithmic and data analytics solutions in the cloud.

The remaining authors declare that the research was conducted in the absence of any commercial or financial relationships that could be construed as a potential conflict of interest.

## Publisher’s note

All claims expressed in this article are solely those of the authors and do not necessarily represent those of their affiliated organizations, or those of the publisher, the editors and the reviewers. Any product that may be evaluated in this article, or claim that may be made by its manufacturer, is not guaranteed or endorsed by the publisher.

## Footnotes

^1^ https://ojs.library.ubc.ca/index.php/UJEMI/issue/view/183016

^2^ https://ojs.library.ubc.ca/index.php/UJEMI/issue/view/183015
