# Supplementary material for: Development of a data science CURE in microbiology using publicly available microbiome datasets
Source: Front Microbiol. 2022 Oct 12;13:1018237. doi: 10.3389/fmicb.2022.1018237 (PMC9597637; doi:10.3389/fmicb.2022.1018237)
Supplement: Supplementary file 1 [file Data_Sheet_1.DOCX]

Supplementary Material

**1 Supplementary Material**

**Rubric used to assess student Project Proposals**

|  | **Clearly meets expectations.**  **Few, if any problems** | **Acceptable but definite weaknesses** | **Does not meet expectations.**  **Significant weaknesses or failed to include** | **Criteria** |
| --- | --- | --- | --- | --- |
| **Proposed Title** | 2 | 1 | 0 | Suitable and relevant to proposal. Sufficient detail to be unique and complete with regard to the objectives. |
| **Introduction and Background** | 6 | 3 | 0 | Clearly written. Addresses details needed to understand the explanation, approach and  outcomes for your proposal. Describes the premise for collection of the dataset and provides a relevant review of already published findings on the dataset. Do not include figures or tables in your introduction. |
| **Research Objectives** | 4 | 2 | 0 | Clearly articulated explanation for the proposed research including research question(s) and corresponding hypothes(es). Hypotheses are not written as predictions. Explain motivation behind study proposal/research question(s). |
| **Experimental Aims** | 4 | 2 | 0 | Clearly written list of research aims that relate to the research objectives (ie. answering your research question(s). |
| **Proposed Approach/**  **Methods** | 8 | 4 | 0 | Provides a Table that describes the “purpose” and “analysis” that will be conducted to achieve each experimental aim. Be specific how the analysis will be conducted (eg. On qiime2 or R, do you need to filter the data first?) |
| **Overview Flow Chart** | 4 | 2 | 0 | Brief overview that shows a diagrammatic relationship of the aims within the context of the research question(s) and how each aim will be approached (eg. method/analysis used). Top to bottom: Research questionà Aims à Approach |
| **Weekly Time Frame for Completing the Work(Gantt Chart)** | 4 | 2 | 0 | Week by week staging of the work. Allow time to test/work with unfamiliar software. Also, allow for time to draft the manuscript. |
| **Feasibility** | 4 | 2 | 0 | Description of the main obstacles or difficulty that might be encountered in the approach.  If you plan to do any analyses not covered in lectures, make references to methods published elsewhere or how you plan to acquire training on those analyses. |
| **References** | 4 | 2 | 0 | At least 6 background references  supporting the proposed explanation and protocol. Proper ASM style. |
| **Participation Report** | 2 | 1 | 0 | Includes a meaningful section describing how each team member contributed to the work and the report. |
| **Overall Impression** | 10 | 5 | 0 | Demonstrates care and critical understanding throughout the proposal. Has been checked to eliminate most spelling, grammar and syntax problems. Clearly and concisely presented. Complete. Readily understood. |

**Rubric used to assess student Manuscripts**

|  | **Consideration** | **Clearly meets expectations** | **Mostly meets expectations but with a few weaknesses** | **Acceptable but with definite weaknesses** | **Poorly meets expectations, significant weaknesses** | **Failed to include or does not meet expectations** | **Criteria** |
| --- | --- | --- | --- | --- | --- | --- | --- |
| **Title** | Clear, accurate, and relevant | 2 | NA | 1 | NA | 0 | Concisely conveys the outcome of project, and is supported by data presented |
| **Abstract** | Clear and accurate. Appropriate format | 2 | 1.5 | 1 | 0.5 | 0 | Easy to read. Consistent with article. Includes purpose, main observations and conclusion(s). No abbreviations or references |
|  | Relevant details | 2 | 1.5 | 1 | 0.5 | 0 |  |
| **Introduction** | Clear and accurate | 2 | NA | 1 | NA | 0 | Easy to follow. Focused on the purpose of project. Proper coverage of background for the analysis and discussion. Does not include parts that should be in other sections |
|  | Relevant details. Appropriate content. | 4 | 3 | 2 | 1 | 0 |  |
|  | Appropriate depth | 4 | 3 | 2 | 1 | 0 |  |
| **Materials and Methods** | Complete and accurate | 2 | NA | 1 | NA | 0 | Mentions relevant details. Uses paragraph format. Uses citations where appropriate. Provides enough detail to follow approach. |
|  | Appropriate format and content. Suitable abbreviations | 2 | 1.5 | 1 | 0.5 | 0 |  |
| **Results** | Consistent and accurate processing | 6 | 4 | 2 | 1 | 0 | Consistent interpretation of data. No processing errors |
|  | Appropriate format. Details are clear and accurate | 10 | 8 | 6 | 4 | 2-0 | Tables and figures formatted with expected details. Figure legend provides necessary information (eg. Statistical analysis performed). |
|  | Appropriate interpretation of the results | 6 | 4 | 2 | 1 | 0 | Interpretation of trends and observations is reasonable |
|  | Appropriate observations | 4 | 3 | 2 | 1 | 0 | Has recognized the major relevant observations |
| **Discussion** | Clear and accurate | 4 | 3 | 2 | 1 | 0 | Statements are consistent with the results, help to understand the results and relate the results to the purpose. Statements show insight. Makes use of supporting knowledge. Covers all major observations |
|  | Relevant | 4 | 3 | 2 | 1 | 0 |  |
|  | Reasonable depth of analysis | 4 | 3 | 2 | 1 | 0 |  |
|  | Study limitations | 4 | 3 | 2 | 1 | 0 | Cover major limitations in the study. Study limitations mentioned are relevant and useful to audience. |
| **Conclusion** | Accurate deductive statement | 4 | 3 | 2 | 1 | 0 | Deductive statement “proved” by the results rather than explanations |
|  | Addresses the experimental question | 4 | 3 | 2 | 1 | 0 | Conclusion addresses the experimental question |
| **Future Directions** | Relevant and feasible | 6 | 4 | 2 | 1 | 0 | Addresses a significant problem or explanation raised in the discussion. Relevant to original purpose. |
|  | Outcomes | 2 | 1.5 | 1 | 0.5 | 0 |  |
| **References** | ASM format, accurate and appropriate citation | 4 | 3 | 2 | 1 | 0 | Cited by number in the manuscript. Correct ASM style in listing. |
| **Supplemental Material** | Script | 2 | NA | 1 | NA | 0 | Annotated and saved as an appropriate file type. |
| **Lab Notebook** | Comprehensive | 2 | NA | 1 | NA | 0 | Detailed record of *in silico* experiments |
|  | Organization | 2 | NA | 1 | NA | 0 | Well organized and structured; easy to follow |
| **Participation Report** | Explain how each author contributed to the project and the report. | 2 | NA | 1 | NA | 0 | Includes a meaningful section describing how each team member contributed to the work and the report. |
| **Overall** | Overall impression | 10 | 8 | 6 | 4 | 2-0 | Demonstrates care and critical understanding throughout the report. Correct language, tenses, and terms. Good insight into results. |

**Prior Experience Survey Questions**

The following questions were asked before the LCAS questions:

Q1. Please summarize your previous research experience(s) outside of this course and classify them based on when you completed the experience. Drag and drop items into the different boxes.

Boxes: 1st year, 2nd year, 3rd year, 4th year, 5th year and above

Q2. Before the course, did you have any data science/bioinformatics training? If yes, please specify (Eg. what course(s), co-op, etc)

Q3a. Did anyone on your team have prior bioinformatics training/experience? If so, how many people?

Q3b. If response to Q3a was Yes: Did you feel that it was helpful to have at least one person on your team with prior bioinformatics training?

Q3c. If response to Q3a was No: Did you feel at a disadvantage not having anyone on your team with prior bioinformatics experience?

**2 Supplementary Table**

**Supplementary Table 1.** Summary of a subset of data science focused undergraduate research courses published since 2008. Educational initiatives have been subdivided into different fields of study that are typically characterized by the biological molecule of interest and/or how it is analyzed.

| **Field of study** | **Description** | **Tool(s)** | **References** |
| --- | --- | --- | --- |
| Genomics | Gene annotation, predicting open reading frames, characterizing miRNAs, predicting mutations, network mapping | BLAST (Altschul et al., 1990)  MAUVE(Darling et al., 2004)  STRING (Szklarczyk et al., 2021) | (Lau and Robinson, 2009; Baumler et al., 2012; Smith et al., 2015; Brown, 2016; Staub et al., 2016; Emery and Morgan, 2017; Achappa et al., 2020) |
| Metagenomics | Statistical comparison of genomes collected from mixed populations of microbes | R (R Core Team, 2022) | (Cottone and Yoon, 2020; Kruchten, 2020) |
| Phylogenetics | Multiple sequence alignment to generate phylogenetic trees | ClustalW (Thompson et al., 1994)  Clustal Omega | (Campo and Garcia-Vazquez, 2008; Furge et al., 2009; Kleinschmit et al., 2019; Achappa et al., 2020) |
| Transcriptomics | Gene expression profiling using RNASeq | DNA Subway (Fast Track to Gene Annotation and Genome Analysis - DNA Subway) and DESeq (Love et al., 2014) | (Makarevitch et al., 2015) |
| Proteomics | Protein profiling through MALDI-TOF MS | Jmol (Jmol: an open-source Java viewer for chemical structures in 3D)  BLASTp | (Furge et al., 2009; Benskin and Chen, 2012) |
| Microbiome | Analysis of microbial diversity collected from amplicon sequencing data | BLAST  QIIME 2 (Bolyen et al., 2019) | (Weber et al., 2018; Sewall et al., 2020; Zelaya et al., 2020; Baker et al., 2021) |
| Metabolomics | Analysis of microbial metabolic pathways | Phylogeny.fr (Phylogeny.fr: Home)  MMDB (Madej et al., 2014) | (Parks and Taylor, 2022) |

Achappa, S., Patil, L., Hombalimath, V., and Shet, A. (2020). Implementation of Project-Based-Learning (PBL) Approach for Bioinformatics Laboratory Course. *J. Eng. Educ. Transform.* 33, 247–252. doi: 10.16920/jeet/2020/v33i0/150154.

Altschul, S. F., Gish, W., Miller, W., Myers, E. W., and Lipman, D. J. (1990). Basic local alignment search tool. *J. Mol. Biol.* 215, 403–410. doi: 10.1016/S0022-2836(05)80360-2.

Baker, S. S., Alhassan, M. S., Asenov, K. Z., Choi, J. J., Craig, G. E., Dastidar, Z. A., et al. (2021). Students in a Course-Based Undergraduate Research Experience Course Discovered Dramatic Changes in the Bacterial Community Composition Between Summer and Winter Lake Samples. *Front. Microbiol.* 12. doi: 10.3389/fmicb.2021.579325.

Baumler, D. J., Banta, L. M., Hung, K. F., Schwarz, J. A., Cabot, E. L., Glasner, J. D., et al. (2012). Using Comparative Genomics for Inquiry-Based Learning to Dissect Virulence of Escherichia coli O157:H7 and Yersinia pestis. *CBE—Life Sci. Educ.* 11, 81–93. doi: 10.1187/cbe.10-04-0057.

Benskin, J., and Chen, S. (2012). Proteomics in the Classroom: An Investigative Study of Proteins in Microorganisms. *Am. Biol. Teach.* 74, 237–243. doi: 10.1525/abt.2012.74.4.6.

Bolyen, E., Rideout, J. R., Dillon, M. R., Bokulich, N. A., Abnet, C. C., Al-Ghalith, G. A., et al. (2019). Reproducible, interactive, scalable and extensible microbiome data science using QIIME 2. *Nat. Biotechnol.* 37, 852–857. doi: 10.1038/s41587-019-0209-9.

Brown, J. A. L. (2016). Evaluating the effectiveness of a practical inquiry-based learning bioinformatics module on undergraduate student engagement and applied skills. *Biochem. Mol. Biol. Educ.* 44, 304–313. doi: 10.1002/bmb.20954.

Campo, D., and Garcia-Vazquez, E. (2008). Inquiry-based learning of molecular phylogenetics. *J. Biol. Educ.* 43, 15–20. doi: 10.1080/00219266.2008.9656144.

Cottone, A. M., and Yoon, S. (2020). Improving the Design of Undergraduate Biology Courses toward the Goal of Retention: The Case of Real-World Inquiry and Active Learning through Metagenomics. *J. Microbiol. Biol. Educ.* 21, 20. doi: 10.1128/jmbe.v21i1.1965.

Darling, A. C. E., Mau, B., Blattner, F. R., and Perna, N. T. (2004). Mauve: Multiple Alignment of Conserved Genomic Sequence With Rearrangements. *Genome Res.* 14, 1394–1403. doi: 10.1101/gr.2289704.

Emery, L. R., and Morgan, S. L. (2017). The application of project-based learning in bioinformatics training. *PLOS Comput. Biol.* 13, e1005620. doi: 10.1371/journal.pcbi.1005620.

Fast Track to Gene Annotation and Genome Analysis - DNA Subway Available at: https://dnasubway.cyverse.org/ [Accessed August 4, 2022].

Furge, L. L., Stevens-Truss, R., Moore, D. B., and Langeland, J. A. (2009). Vertical and horizontal integration of bioinformatics education: A modular, interdisciplinary approach. *Biochem. Mol. Biol. Educ. Bimon. Publ. Int. Union Biochem. Mol. Biol.* 37, 26–36. doi: 10.1002/bmb.20249.

Jmol: an open-source Java viewer for chemical structures in 3D Available at: http://jmol.sourceforge.net/#cite [Accessed August 4, 2022].

Kleinschmit, A., Brink, B., Roof, S., Goller, C. C., and Robertson, S. (2019). Sequence Similarity: An inquiry based and “under the hood” approach for incorporating molecular sequence alignment in introductory undergraduate biology courses. *CourseSource*. doi: 10.25334/Q4G45Z.

Kruchten, A. E. (2020). A Curricular Bioinformatics Approach to Teaching Undergraduates to Analyze Metagenomic Datasets Using R. *Front. Microbiol.* 11, 578600. doi: 10.3389/fmicb.2020.578600.

Lau, J. M., and Robinson, D. L. (2009). Effectiveness of a cloning and sequencing exercise on student learning with subsequent publication in the National Center for Biotechnology Information GenBank. *CBE Life Sci. Educ.* 8, 326–337. doi: 10.1187/cbe.09-05-0036.

Love, M. I., Huber, W., and Anders, S. (2014). Moderated estimation of fold change and dispersion for RNA-seq data with DESeq2. *Genome Biol.* 15, 550. doi: 10.1186/s13059-014-0550-8.

Madej, T., Lanczycki, C. J., Zhang, D., Thiessen, P. A., Geer, R. C., Marchler-Bauer, A., et al. (2014). MMDB and VAST+: tracking structural similarities between macromolecular complexes. *Nucleic Acids Res.* 42, D297–D303. doi: 10.1093/nar/gkt1208.

Makarevitch, I., Frechette, C., and Wiatros, N. (2015). Authentic Research Experience and “Big Data” Analysis in the Classroom: Maize Response to Abiotic Stress. *CBE Life Sci. Educ.* 14, ar27. doi: 10.1187/cbe.15-04-0081.

Parks, S. T., and Taylor, C. (2022). Development of a Remote, Course-Based Undergraduate Experience to Facilitate In Silico Study of Microbial Metabolic Pathways. *J. Microbiol. Biol. Educ.* 23, e00318-21. doi: 10.1128/jmbe.00318-21.

Phylogeny.fr: Home Available at: https://www.phylogeny.fr/ [Accessed August 4, 2022].

R Core Team (2022). R: A language and environment for statistical computing. *R Found. Stat. Comput. Vienna Austria*. Available at: https://www.R-project.org/.

Sewall, J. M., Oliver, A., Denaro, K., Chase, A. B., Weihe, C., Lay, M., et al. (2020). Fiber Force: A Fiber Diet Intervention in an Advanced Course-Based Undergraduate Research Experience (CURE) Course †. *J. Microbiol. Biol. Educ.* 21. doi: 10.1128/jmbe.v21i1.1991.

Smith, J. T., Harris, J. C., Lopez, O. J., Valverde, L., and Borchert, G. M. (2015). “On the job” learning: A bioinformatics course incorporating undergraduates in actual research projects and manuscript submissions. *Biochem. Mol. Biol. Educ. Bimon. Publ. Int. Union Biochem. Mol. Biol.* 43, 154–161. doi: 10.1002/bmb.20848.

Staub, N. L., Poxleitner, M., Braley, A., Smith-Flores, H., Pribbenow, C. M., Jaworski, L., et al. (2016). Scaling Up: Adapting a Phage-Hunting Course to Increase Participation of First-Year Students in Research. *CBE Life Sci. Educ.* 15, ar13. doi: 10.1187/cbe.15-10-0211.

Szklarczyk, D., Gable, A. L., Nastou, K. C., Lyon, D., Kirsch, R., Pyysalo, S., et al. (2021). The STRING database in 2021: customizable protein-protein networks, and functional characterization of user-uploaded gene/measurement sets. *Nucleic Acids Res.* 49, D605–D612. doi: 10.1093/nar/gkaa1074.

Thompson, J. D., Higgins, D. G., and Gibson, T. J. (1994). CLUSTAL W: improving the sensitivity of progressive multiple sequence alignment through sequence weighting, position-specific gap penalties and weight matrix choice. *Nucleic Acids Res.* 22, 4673–4680.

Weber, K. S., Bridgewater, L. C., Jensen, J. L., Breakwell, D. P., Nielsen, B. L., and Johnson, S. M. (2018). Personal microbiome analysis improves student engagement and interest in Immunology, Molecular Biology, and Genomics undergraduate courses. *PLOS ONE* 13, e0193696. doi: 10.1371/journal.pone.0193696.

Zelaya, A. J., Gerardo, N. M., Blumer, L. S., and Beck, C. W. (2020). The Bean Beetle Microbiome Project: A Course-Based Undergraduate Research Experience in Microbiology. *Front. Microbiol.* 11. doi: 10.3389/fmicb.2020.577621.

# **3 Supplementary Figures**


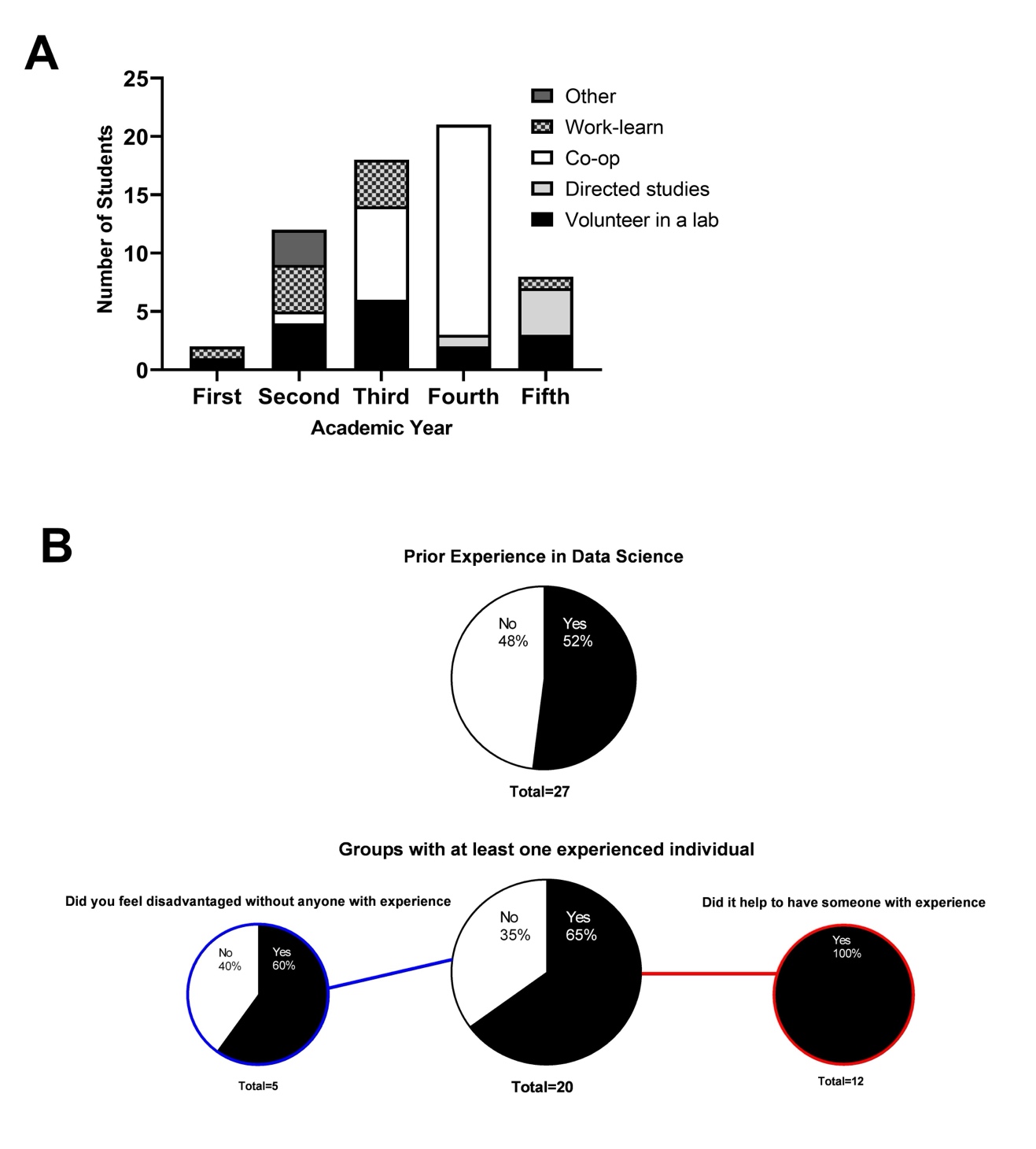


**Supplementary Figure 1.** Prior undergraduate research and data science experience in student cohort undertaking the data science CURE. This survey was implemented in Sept-Dec 2020 (60 students) and Jan-Apr 2021 (18 students). There was a response rate of 25-35% depending on the question. (A) n=27, (B) n=27 for question about prior data science experience and n=20 in regards to having at least one experienced individual in their team.
